# The mRNA Expression Status of Dopamine Receptor D2, Dopamine Receptor D3 and DARPP-32 in T Lymphocytes of Patients with Early Psychosis

**DOI:** 10.3390/ijms161125983

**Published:** 2015-11-06

**Authors:** Yin Cui, Vishwanath Prabhu, Thong Ba Nguyen, Binod Kumar Yadav, Young-Chul Chung

**Affiliations:** 1 Department of Psychiatry, Chonbuk National University Medical School, Jeonju 561-756, Korea; cuiyin61@jbnu.ac.kr (Y.C.); vvprabhu@jbnu.ac.kr (V.P.); 201555433@jbnu.ac.kr (T.B.N.); binod3aug@jbnu.ac.kr (B.K.Y.); 2 Research Institute of Clinical Medicine of Chonbuk National University-Biomedical Research Institute of Chonbuk National University Hospital, Jeonju 561-756, Korea

**Keywords:** early psychosis, T lymphocytes, mRNA, *DRD2*, *DRD3*, *DARPP-32*

## Abstract

Peripheral blood lymphocytes are an attractive tool because there is accumulating evidence indicating that lymphocytes may be utilized as a biomarker in the field of psychiatric study as they could reveal the condition of cells distributed in the brain. Here, we measured the mRNA expression status of *dopamine receptor D2 (DRD2)*, *DRD3*, and *dopamine and cyclic adenosine 3′,5′-monophosphate regulated phosphoprotein-32 (DARPP-32)* in T lymphocytes of patients with early psychosis by quantitative real-time polymerase chain reaction (q-PCR) and explored the relationships between their mRNA levels and the psychopathological status of patients. The present study demonstrated that the mRNA expression levels of *DRD3* in T lymphocytes were significantly different among controls, and in patients with psychotic disorder not otherwise specified (NOS) and schizophrenia/schizophreniform disorder. However, no significant differences in mRNA expression levels of *DRD2* and *DARPP-32* were found among the three groups. We found a significant positive correlation between the *DRD2* mRNA level and the score of the excited factor of the Positive and Negative Syndrome Scale (PANSS) in patients with schizophrenia/schizophreniform disorder. These findings suggest that *DRD3* mRNA levels may serve as a potential diagnostic biomarker differentiating patients with early psychosis from controls.

## 1. Introduction

Schizophrenia is a most grave disease among serious mental illnesses. Although the annual incidence is very low at 15.2 cases per 100,000 persons/year [1], it often has a devastating impact on patients’ quality of life: about two-thirds of the subjects with schizophrenia take a chronic course with relapse [2,3] and schizophrenic patients have two-fold to three-fold higher mortality rates compared to the general population, corresponding to a 10- to 25-year reduction in life expectancy [4]. Current diagnostic approaches for schizophrenia are solely based on patient interviews, which entail a subjective assessment of clinical symptoms. The accuracy of existing diagnostic methods can be problematic when subjects often show significant overlap of symptoms with those displayed by individuals affected by other psychiatric conditions such as bipolar disorder and major depressive disorder. This inaccuracy is especially apparent in the individuals with the “premorbid” phase of schizophrenia or attenuated psychosis syndrome. These problems may indicate that a diagnostic biomarker differentiating schizophrenia from other major mental illnesses, especially in the early stage of the disease, may have significant implications.

With rapid technological advances in neuroscience, vigorous research using brain imaging technologies is being conducted to find biomarkers for schizophrenia. Though several interesting findings are suggested as biomarkers for psychosis onset, treatment response, or relapse [5,6,7], this approach is often difficult in terms of cost, time, and accessibility. On the other hand, blood-based biomarkers have greater advantages in those regards and may enable a simpler and more rapid diagnosis and monitoring of prognosis and outcome for schizophrenia. Especially peripheral blood lymphocytes are an attractive tool because there is accumulating evidence indicating that lymphocytes may be utilized as a biomarker in the field of psychiatric study as they could reveal the condition of cells distributed in the brain [8]. Studies have suggested that lymphocytes are capable of producing dopamine (DA) [9,10] and express the rate-limiting enzyme tyrosine hydroxylase [11]. Moreover, the expression of dopamine receptor (DR) *D2*–*D5* genes, but not the *DRD1* gene, in lymphocytes of the human peripheral blood mononuclear cells (PBMCs) fraction was measured by quantitative real-time polymerase chain reaction (RT-PCR) [12] and flow cytometry [13]. As the DR in PBMCs may reveal the status of homologous brain receptors [14,15] and the DA hypothesis is still the dominant theory of schizophrenia, there have been several studies examining expression levels of DR in the PBMCs of patients with schizophrenia. Although more studies are needed to reach a definite conclusion, the increased expression of *DRD3* mRNA in the lymphocytes of schizophrenic patients was suggested as a diagnostic marker for schizophrenia [16,17,18,19]. It should be noted that, in most of previous studies, PBMCs, not specific subpopulations of lymphocytes, were used, and the duration of illness (DI) of the participants was long or not specified. Using RT-PCR, *DRD3* was only found in T cells and natural killer cells [16]. The relative proportion of T cells accounts for about 75% of all lymphocytes [20]. Given that the homogenous expression of DR on all different subpopulation cells cannot be assumed, we hypothesized that the investigation of the expression of DR genes in specific cells, especially T lymphocytes, would give more consistent results. In addition, recruiting patients with early psychosis (DI ≤ 5 years) would be desirable because accurate diagnosis in this stage is crucially important. Psychotic disorder not otherwise specified (NOS) may be used in diagnosis when there is inadequate/contradictory information to make a specific diagnosis or psychotic symptoms do not meet the criteria for any specific psychotic disorder Diagnostic and Statistical Manual of Mental Disorders (Fourth Edition) (DSM-IV) [21]. In terms of the psychosis continuum theory, it may represent the middle ground between normal and schizophrenia. Hence, it would be also interesting to divide the patients with early psychosis into patients with psychotic disorder NOS and schizophrenia/schizophreniform disorder and compare the results. The aims of this study were to identify the mRNA expression status of *DRD2*, *DRD3*, and *dopamine and cyclic adenosine 3′,5′-monophosphate regulated phosphoprotein-32 (DARPP-32)* in T lymphocytes of patients with early psychosis, and to explore the relationships between the mRNA levels of the three targeted genes and the psychopathological status of patients evaluated by the Positive and Negative Syndrome Scale (PANSS) [22].

## 2. Results and Discussion

### 2.1. Results

As for the demographic characteristics of the subjects (Table 1), there were no significant differences in age and sex among the three groups. The DI of psychotic disorder NOS and schizophrenia/schizophreniform disorder was 27.26 ± 34.77 months and 22.91 ± 15.06 months, respectively. Nineteen subjects were on medication at the time of blood sampling and their mean dose of chlorpromazine equivalency was 438.61 ± 288.91 mg/day*.*

**Table 1:** Demographic characteristics of the subjects.

Characteristic	Control (*n* = 30)	Psychotic Disorder NOS (*n* = 18)	Schizophrenia/Schizophreniform Disorder (*n* = 14)	Medicated Group (*n* = 19)	Drug-naive/Drug-free Group (*n* = 13)
Mean age (SD)	30.03 (8.35)	29.61 (13.24)	36.50 (12.95)	31.63 (12.01)	34.25 (16.20)
Sex, m/f	14/16	9/9	6/8	9/10	6/7

#### 2.1.1. Comparison of *DRD2*, *DRD3*, and *DARPP-32* mRNA Levels among Controls, and Patients with Psychotic Disorder NOS and Schizophrenia/Schizophreniform Disorder

**Figure 1:**
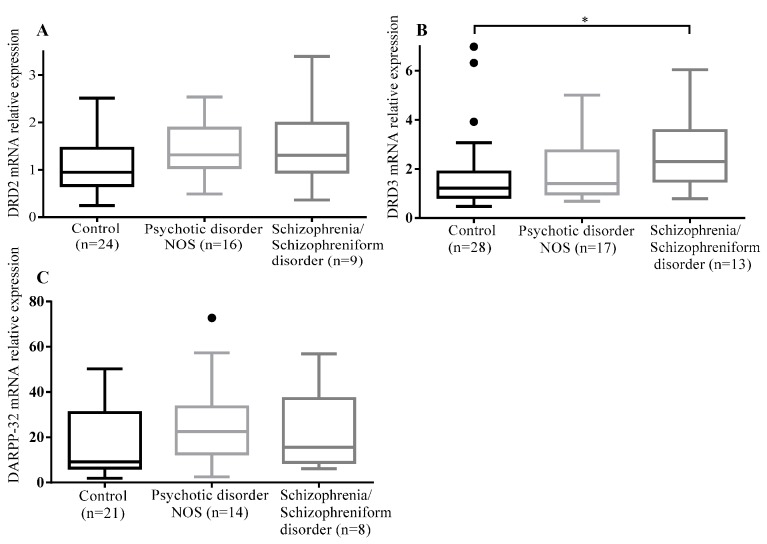
Comparison of *DRD2*, *DRD3*, *DARPP-32* mRNA levels among the three groups: (**A**) *DRD2* mRNA levels among the three groups; (**B**) *DRD3* mRNA levels among the three groups; and (**C**) *DARPP-32* mRNA levels among the three groups. Significant difference was only observed in the mRNA expression levels of *DRD3* in T lymphocytes among the three groups (* *p* = 0.03). The box plots display the median and interquartile range (25th to 75th percentiles), and the whiskers outside the box extend to the highest and lowest value within 1.5 times the interquartile range. Points outside the whiskers are outliers.

The mRNA expression levels of *DRD3* in T lymphocytes were significantly different among controls and patients with psychotic disorder NOS and schizophrenia/schizophreniform disorder (*p* = 0.03). The post-hoc analysis with Dunn’s multiple comparison test showed that the mRNA expression level of DRD3 in T lymphocytes of patients with schizophrenia/schizophreniform disorder was significantly greater than that of the controls (*p* = 0.03). However, no significant differences in mRNA expression levels of *DRD2* and *DARPP-32* were found among three groups (Table 2 and Figure 1).

**Table 2:** Comparison of dopamine receptor D2 (DRD2), dopamine receptor D3 (DRD3), and dopamine and cyclic adenosine 3′,5′-monophosphate regulated phosphoprotein-32 (DARPP-32) mRNA levels among controls and patients with psychotic disorder NOS and schizophrenia/schizophreniform disorder (median (interquartile range)).

Gene	Control (n)	Psychotic Disorder NOS (n)	Schizophrenia/Schizophreniform Disorder (n)	*p*-Value
*DRD2*	0.95 (0.67–1.45) (24)	1.32 (1.05–1.87) (16)	1.31 (0.95–1.98) (9)	0.19
*DRD3*	1.22 (0.85–1.88) (28)	1.41 (0.99–2.75) (17)	2.30 (1.50–3.57) (13) ^†^	0.03 *
*DARPP-32*	9.21 (6.38–30.93) (21)	22.48 (12.77–33.46) (14)	15.64 (8.92–37.08) (8)	0.31

* Significantly different among three groups; ^†^ Significantly different between patients with schizophrenia/schizophreniform disorder and controls using Dunn’s multiple comparison test.

#### 2.1.2. Comparison of *DRD2*, *DRD3* and *DARPP-32* mRNA Levels among Controls, and Drug-Naive/Drug-Free and Medicated Patients

The mRNA expression levels of *DRD2*, *DRD3*, and *DARPP-32* were not significantly different among the three groups (Table 3).

**Table 3:** Comparison of *DRD2*, *DRD3*, and *DARPP-32* mRNA levels among controls and drug-naive/drug-free and medicated patients (median (interquartile range)).

Gene	Control (n)	Drug-Naive/Drug-Free Group (n)	Medicated Group (n)	*p* Value
*DRD2*	0.95 (0.67–1.45) (24)	1.18 (0.94–2.03) (10)	1.35 (1.12–1.79) (15)	0.19
*DRD3*	1.22 (0.85–1.88) (28)	1.78 (0.97–4.39) (12)	1.88 (1.22–2.89) (18)	0.08
*DARPP-32*	9.21 (6.38–30.93) (21)	18.52 (12.86–33.46) (10)	19.61(8.82–36.34) (12)	0.30

#### 2.1.3. Correlation Analysis between *DRD2*, *DRD3*, and *DARPP-32* mRNA Levels and Five Factors of the PANSS in Patients with Psychotic Disorder NOS and Schizophrenia/Schizophreniform Disorder

There was a significant positive correlation between *DRD2* mRNA levels and scores of the excited factor of the PANSS in patients with schizophrenia/schizophreniform disorder according to Spearman’s correlation test (*r* = 0.616, *p* = 0.04) (Table 4).

**Table 4:** Spearman rank correlations analysis between *DRD2*, *DRD3*, and *DARPP-32* mRNA levels and five factors of the PANSS in patients with psychotic disorder NOS and the schizophrenia/schizophreniform disorder group.

PANSS Factors	Psychotic Disorder NOS			Schizophrenia/Schizophreniform Disorder		
	DRD2	DRD3	DARPP-32	DRD2	DRD3	DARPP-32
Positive	−0.296	−0.040	0.147	0.487	−0.061	0.146
Cognitive	−0.287	0.043	−0.157	0.393	0.092	0.254
Negative	−0.338	0.341	0.077	0.189	−0.058	0.244
Excited	−0.396	−0.065	0.176	0.616 *	0.074	−0.405
Anxiety/Depression	−0.093	−0.076	0.252	0.326	0.292	−0.319

* *p*-value = 0.04.

### 2.2. Discussion

Biomarkers are now applied in many fields of medicine. However, their application in the area of psychiatry is rare. This study was undertaken to explore peripheral blood-based biomarkers that could distinguish individuals with early psychosis from controls. As a result, we found that *DRD3* mRNA expression levels were significantly different between subjects with early psychosis and controls.

The question of whether PBMCs can be used as surrogate markers for the brain has elicited a lot of debate. Recently, Rollins *et al.* [15] reported a very high correlation (0.98) of gene expression between the matched pairs of postmortem PBMCs and cerebellum in a subset (22%) of the total transcriptome (4103 genes of 17,859 genes). These results support the feasibility of probing genes related to neural function in the peripheral blood transcriptome. On the other hand, Kirillova *et al.* [12] demonstrated that mRNA expression levels of *DRD3* and *DRD4* in PBMCs are comparable to those in the brain, whereas mRNA expression levels of *DRD2* and *DRD5* are notably lower. They suggested that DR expression in lymphocytes is not reflective of that in the brain and low and variable expression of the DR may explain the inconsistent results with isolated PBMCs in patients with psychiatric disorders. Despite the controversy, PBMCs or lymphocytes are certainly an easily accessible sample that may serve as a neural and genetic probe to investigate mental illnesses in living individuals. One important issue in the study of blood-based biomarkers is whether to use whole PBMCs or specific cell subsets. Since subpopulations of PBMCs are functionally different and the expression of DR in diverse cell subsets is varied [13,16], different samples may produce conflicting results. From this aspect, the strength of the present study lies in the use of specific T lymphocytes. Up until now, there were only two studies examining DR expression levels in patients with schizophrenia using T lymphocytes: one study reported increased percentages of CD8^+^D2^+^ cells [23] and the other revealed a significantly higher expression of *DRD3* mRNA [16].

As for the *DRD2* mRNA level, we did not find a significant difference among the three groups which is consistent with the findings of other studies [19,24,25]. However, two studies reported the increased expression of *DRD2* in patients with schizophrenia; in the Zvara *et al.* [26] study, PBMCs were isolated from drug-naïve or drug-free subjects with a varied duration of illness and severe symptoms (PANSS total score was 119.07 ± 22.57), and in the Brito-Melo *et al.* [23] study, T cell subsets (CD4 and CD8) from medicated (dose of chlorpromazine equivalent to 480.70 ± 257.80 mg/day) chronic patients with DI > 10 years were used. It is of interest to note that loxapine treatment down-regulated lymphocyte *D2*-like receptors in patients with schizophrenia (mean age of 39 years) [27]. Considering that patients in the present study were showing mild symptoms (PANSS total score was 61.81 ± 22.50) and 19 were medicated, the negative result may be due to the difference of symptom severity or medication effect. When we re-analyzed the results by dividing the subjects into non-medicated (drug-naïve and drug-free) and medicated groups, no difference in the *DRD2* expression level was found. As a main finding of our study, a significant difference in *DRD3* mRNA levels was found among controls and patients with psychotic disorder NOS and schizophrenia/schizophreniform disorder. The result of increased expression of *DRD3* mRNA in T lymphocytes of patient groups is in line with results of previous studies [16,17,18,19], whereas no change [28] or decreased [29] expression of *DRD3* mRNA was also reported. Given that subjects showing increased expression of *DRD3* mRNA compared with controls are either drug-free and/or drug-naïve [17,19] or all or mostly medicated [16,18], it seems that the increased expression of *DRD3* mRNA is a consistent finding regardless of medication status. The implication of *DRD3* in the pathophysiology of schizophrenia has been supported by multiple lines of evidence. First, *DRD3* is preferentially localized in limbic areas, suggesting its role in emotions, motivation, and reward. Higher levels of *DRD3* mRNA were expressed in the nucleus accumbens and islands of Calleja with relatively lower levels of expression in the anterior caudate and putamen in the human brain [30]. Second, the overexpression of the *DRD3* has been suggested as a mechanism for behavioral sensitization in a rodent model of Parkinson’s disease [31,32]. Third, an elevated *DRD* receptor level was observed in the brains of drug-free schizophrenic patients, but not in medicated patients at the time of death [33]. Assuming that the increased expression of *DRD3* mRNA in T lymphocytes may reflect hyperdopaminergic neurotransmission in the brain, this finding can be interpreted to show that hyperdopaminergic function induced by *DRD3* may exist in patient groups and *DRD3* mRNA is a diagnostic biomarker for early psychosis. Furthermore, it is interesting to note that the level of *DRD3* mRNA in patients with psychotic disorder NOS was between that of the controls and patients with schizophrenia/schizophreniform disorder. This finding supports a continuum model of psychosis [34], though it needs to be reconfirmed in future studies with large sample sizes. As for the *DARPP-32* mRNA level, no significant difference was found among the three groups, which is in contrast with the findings of the Torres *et al.* [35] study showing that *DARPP-32* expression was decreased in CD4^+^ T lymphocytes and CD56^+^ natural killer cells of schizophrenic patients. As the subjects in the Torres *et al.* [35] study were old and mostly exposed to high doses of antipsychotics, direct comparison should be done with caution. *DARPP-32* plays an important role in the transduction of dopaminergic signaling, integrating information from multiple converging pathways in neurons [36]. Hence, this finding indicates that there is no change in intracellular dopaminergic signaling measured in T lymphocytes of patients with early psychosis. The correlation analysis revealed that *DRD2* mRNA levels were positively related to the score of the excited factor of the PANSS. This result is similar to that of the Liu *et al.* [24] study in which a positive correlation between *DRD2* mRNA levels in PBMCs and the positive symptom subscale scores of the PANSS in acute schizophrenia was demonstrated. However, this is in contrast with other findings that the Brief Psychiatric Rating Scale and negative symptom subscale scores of the PANSS were positively related to CD8^+^D2^+^ cells of schizophrenic patients [23]. The implication of this finding may be that the *DRD2* mRNA level in T lymphocytes reflects symptom severity related to the excited factor of the PANSS. In future studies, it would be interesting to investigate whether the *DRD2* mRNA level may change with treatment over time.

Several limitations of the present study need to be mentioned. First, we cannot exclude the possibility that the positive finding in the *DRD3* mRNA level might have been due to a medication effect since 19 of the participants were on medication at the time of blood sampling. Little is known about the effects of antipsychotics on the expression and regulation of DR in the lymphocytes and brain. Kwak *et al.* [17] reported that, after taking antipsychotics, the highest mRNA level of dopamine receptors appeared during the second week and then it decreased, but the level at the eighth week was above baseline. The administration of loxapine has been reported to induce the down-regulation of *D2*-like receptors in lymphocytes of patients with schizophrenia [23]. The administration of haloperidol and clozapine induced down-regulation and up-regulation of *DRD2* mRNA, respectively, in the rat hippocampus while minimal effects on *DRD3* mRNA were demonstrated by both drugs. To address this issue properly, more strict and prudent prospective study design is necessary. Second, because of limited sample amount, we could not confirm whether there are similar changes in protein levels. As a gene’s mRNA level does not usually predict its protein level [37,38], further investigation is warranted. Lastly, the sample size was relatively small, especially for the *DARPP-32* mRNA level. In conclusion, the expression levels of *DRD3* mRNA in T lymphocytes were significantly different among controls and patients with psychotic disorder NOS and schizophrenia/schizophreniform disorder. The *DRD2* mRNA levels were correlated with symptom severity related to the excited factor of the PASNSS in patients with schizophrenia/schizophreniform disorder. These findings suggest that the *DRD3* mRNA level may serve as a potential diagnostic biomarker differentiating patients with early psychosis from controls.

## 3. Experimental Section

### 3.1. Subjects

We screened 40 eligible patients from the inpatient and outpatient unit of the Department of Psychiatry, Chonbuk National University Hospital, between May 2014 and April 2015. The inclusion criteria were subjects who (a) were currently diagnosed with a schizophrenia spectrum disorder (schizophrenia, schizoaffective disorder, schizophreniform disorder, psychotic disorder NOS, brief psychotic disorder) with DI ≤ 5 years confirmed by the Structural Clinical Interview from the Diagnostic and Statistical Manual of Mental Disorders, Fourth Edition (SCID-IV) [39,40]; (b) were aged 16–68 years; and (c) were able to comprehend the procedure and aims of the study. We included subjects with attenuated psychosis syndrome (APS) in the category of psychotic disorder NOS. For the APS, the criteria in the DSM-5 was applied. The exclusion criteria were subjects who (a) were diagnosed with mental retardation (IQ ≤ 70); (b) had a past history of head trauma; (c) had a serious neurologic disorder (epilepsy, stroke, Parkinson’s disease, dementia); and (d) had acute, unstable, significant medical illness. In total, 32 patients were recruited. Sociodemographic (age, sex, marital status, education) and clinical (DI, medication, age of onset) data were obtained. Psychiatric symptom severity was measured with the Positive and Negative Syndrome Scale [22]. Thirty-one age- and sex-matched healthy volunteers were also recruited via advertisement. They were interviewed with the screening module of the SCID-IV, non-patient edition [39,40]. Participants were excluded if they had any of the following: a current or past diagnosis of a psychiatric or neurological disorder, alcohol or drug abuse or dependence (except for nicotine), and other significant medical conditions. All participants provided written informed consent in accordance with a protocol approved by the hospital’s ethics committee (approval number 2014-06-022).

### 3.2. Preparation of T Lymphocytes

Ten mL of fasting ulnar vein blood were drawn from each subject and collected in vacutainer tubes containing preservative-free heparin. PBMCs were isolated by standard density centrifugation (2500 rpm and 25 °C for 30 min without break) using Ficoll-Paque PLUS (d = 1.077, Amersham Pharmacia Biotech, Uppsala, Sweden). Residual red blood cells were completely removed using the red blood cell lysis buffer (BioLegend, San Diego, CA, USA). Platelets were also removed by centrifugation at 900 rpm for 15 min at room temperature. Finally, T lymphocytes were purified using a T cell enrichment column (R&D Systems, Minneapolis, MN, USA) and stored at −70 °C until required for use. About 5 × 10^6^ T cells were obtained from 10 mL whole blood. Purity of T lymphocytes was >92%, evaluated by flow cytometry (BD Biosciences, San Jose, CA, USA) analysis after staining the T lymphocytes with monoclonal antibodies detecting human CD3 Antigens PE (BD Biosciences). Separation of lymphocytes was done withinfour hours after drawing blood.

### 3.3. RNA Extraction, cDNA Synthesis and Quantitative Real-Time Polymerase Chain Reaction (RT-PCR)

Extraction of total ribonucleic acid (RNA) was performed with the TRIzol^®^ Reagent (Life Technology, Carlsbad, CA, USA) as the manual described. Quality and quantity of total RNA were determined by the Colibri Microvolume Spectrophotometer (Titertek-Berthold, Pforzheim, Germany) and ratio of optical density at 260 to 280 nm; 1.8–2.1 was considered acceptable. The total RNA was reverse-transcripted into the first cDNA with PrimeScript™ RT Reagent Kit (TaKaRa Bio, Otsu, Japan). The react system contains total RNA 5× PrimeScript™ Buffer, PrimeScript™ RT Enzyme Mix I, OligodT Primer, Random 6 mers, and RNase Free dH2O. The reacting condition was 37 °C for 15 min, 85 °C for 5 s. The qPCR was conducted by the Applied Biosystems 7900HT Fast Real Time PCR System (Applied Biosystems, Foster City, CA, USA). The reaction system contains cDNA, 1× Power SYBR Green PCR Master Mix (Applied Biosystems, Warrington, UK) and primer (Bioneer Inc., Seoul, Korea) as shown in Table 5, and conditions for all genes were 95 °C for 15 min for pre-denaturation, 94 °C for 15 s for denaturation, 58 °C for 30 s for annealing, 72 °C for 30 s for extension, and cycling for 40 laps. Expressions of DRD2, DRD3, and DARPP-32 were normalized to β-actin and calibrator (the sample loaded in the first well of the platelet) and expressed in arbitrary units using the following formula: R = 2^−ΔΔ*C*t^ (ΔΔ*C*_t_ = Δ*C*_t_ Sample − Δ*C*_t_ calibrator, Δ*C*_t_ = *C*_t_ Target gene − *C*_t_ β-actin).

**Table 5:** The primer sequences of *DRD2*, *DRD3*, *DARPP-32*, and *β-Actin*.

Gene	Accession Code	Sequence	Size (bp)
*DRD2*	NM_000795	For: 5′-AGACCATGAGCCGTAGGAAG-3′	96
		Rev: 5′-GCAGCCAGCAGATGATGA-3′	
*DRD3*	NM_000796	For: 5′-CAACCCTGATTTTGTCATCTACTCT-3′	102
		Rev: 5′-CTTTGTTTCAGCACCACATAGATTC-3′	
*DARPP-32*	NM_032192	For: 5′-CTACACACCACCTTCGCTGA-3′	131
		Rev: 5′-TCTGAGGCCTGGTTCTCATT-3′	
*β-Actin*	NM_001101	For: 5′-GAGCGGGAAATCGTGCGTGACATT-3′	76
		Rev: 5′-GAAGGTAGTTTCGTGGATGCC-3′	

### 3.4. Statistical Analysis

Statistical analysis was performed using statistical software SPSS version 21 (IBM, New York, NY, USA). For demographic data, one-way ANOVA and the Chi-square test were employed. As the data on mRNA levels was not normally distributed, non-parametric tests were employed. The Kruskal-Wallis test was performed to compare expression levels of *DRD2*, *DRD3*, and *DARPP-32* mRNA among controls and patients with psychotic disorder and schizophrenia/schizophreniform disorder. The post-hoc analysis was performed using Dunn’s test with dBSTAT version 5.0(dBSTAT version 5.0, dBSTAT, Seoul, Korea). Spearman’s correlation analysis was used to analyze the relationship between the mRNA levels of the three target genes and the five factor scores of the PANSS [22]. A criterion of *p* < 0.05 was set for statistical significance.

## 4. Conclusions

The expression levels of *DRD3* mRNA in T lymphocytes were significantly different among controls and patients with psychotic disorder NOS and schizophrenia/schizophreniform disorder. The *DRD2* mRNA level was correlated with symptom severity related to the excited factor of the PANSS in patients with schizophrenia/schizophreniform disorder. These findings suggest that the *DRD3* mRNA level may serve as a potential diagnostic biomarker differentiating patients with early psychosis from controls.
